# Smoking Among High School Students in Dili, Timor-Leste: Prevalence, Potential Determinants and Opportunities for Prevention and Control

**DOI:** 10.1177/10105395231173743

**Published:** 2023-05-06

**Authors:** João S. Martins, Susan McAllister, Livio da Conceição Matos, Natalia Pereira, Frederico B. A. dos Santos, Richard Edwards

**Affiliations:** 1 Universidade Nacional Timor Lorosa’e, Díli, Timor Leste; 2 Centre for International Health, University of Otago, Dunedin, New Zealand; 3 School of Midwifery, Faculty of Medicine and Health Sciences, Universidade Nacional Timor Lorosa’e, Díli, Timor Leste; 4 Department of Non-Communicable Diseases, Ministry of Health, Díli, Timor-Leste; 5 Department of Public Health, University of Otago, Wellington, New Zealand

**Keywords:** tobacco, smoking, adolescents, prevalence, exposures

## Abstract

Smoking initiation is concentrated among young people which strongly influences future smoking prevalence. This study aimed to investigate the prevalence of smoking and other tobacco product use and potential determinants in a cross-sectional survey of 1 121 students aged 13 to 15 years in Dili, Timor-Leste. The prevalence of *ever* using a tobacco product was 40.4% (males 55.5%; females 23.8%) and of current use was 32.2% (males 45.3%; females 17.9%). In a logistic multivariable regression, factors associated with current use of any tobacco product were being male, ≥US$1 weekly pocket money, parents smoking, exposure at home, and exposure in other locations. The findings suggest that reducing the very high use of tobacco among adolescents in Timor-Leste will require new policy measures, enhanced enforcement of current legislation as well as a focused commitment to targeted smoke-free education campaigns, and community-based health promotion to support parents to quit smoking and not smoke around children.

## What We Already Know

- The number of people who smoke tobacco remains high across the globe.
- Smoking initiation is concentrated among young people which strongly influences future smoking prevalence.
- Legislations and frameworks to prevent smoking and other tobacco product use have been in place in Timor-Leste for several years.

## What This Article Adds

- There continues to be a high smoking prevalence of tobacco product use and smoking in adolescents in Timor-Leste, particularly amongst boys.
- Specific risk factors associated with tobacco product use and smoking in adolescents identified included parental smoking, exposure to smoking in the home and other public locations, exposure to smoking in the media or messaging about smoking and, having access to pocket money.
- Despite existing anti-smoking legislation in Timor-Leste, stronger enforcement of this legislation needs to be actioned and new policy measures and interventions considered to reduce tobacco product use and smoking among adolescents.

## Introduction

The harmful effects of smoking on people’s health, and those around them from secondhand smoke, have been well documented.^1,2^ However, global prevalence of tobacco smoking remains high (22.3% in 2020).^3^ Prevalence is higher in men (36.7%) than women (7.8%), and an estimated 80% of tobacco users live in low- and middle-income countries.^3^ Smoking intitiation is concentrated among adolescents and young adults with an estimated 83% of all smokers who begin smoking between the ages of 14 and 25.^4,5^ This early initiation strongly influences future smoking prevalence and sustains the smoking epidemic. Preventing smoking initiation in young people is therefore critical to controlling the epidemic.^4,6^

While the prevalence of smoking in young people has largely been declining across the globe,^4,7^ in several Southeast Asian countries, it remains high.^7,8^ Timor-Leste, a small country of 1.3 million people located in the Southeast Asian region, is one such country experiencing high rates of tobacco product use in both young people and adults. The 2013 Global Youth Tobacco Survey (GYTS)—a nationally representative school-based survey reported 42% of adolescents aged 13 to 15 years (66% boys; 24% girls) were consuming tobacco products, and 29% (54% boys; 11% girls) were classified as current cigarette smokers—smoking at least once in the last month.^9^

In 2005, The World Health Organization (WHO) brought into force the Framework Convention on Tobacco Control (FCTC).^10^ Some of the key interventions in the Framework include smoke-free laws and policies to protect people from exposure to tobacco smoke, increasing tobacco excise taxes, education and warnings about the harms from smoking, and restrictions on tobacco advertising—all of which may help decrease the risk of smoking uptake in young people.^11^ Timor-Leste, along with 40 other States and jurisdictions, accepted and ratified the FCTC. In 2015, The Dili Declaration on Tobacco Control was also signed which listed 10 commitments for the prevention and control of tobacco in the region.^12^ Since the FCTC and the Declaration, the government has prohibited smoking in many indoor public places, workplaces, and on public transportation; prohibited many forms of tobacco advertising and promotion—except for point-of-sale product displays; introduced very large pictorial health warnings on cigarette packaging; and prohibited the sale of single and small packets of cigarettes, sales to persons under the age of 17, and sales in educational facilities, playgrounds, stadiums, health care institutions and other specified locations.^13^ However, high smoking rates in youth persist, with a 2019 GYTS reporting 30.9% of students aged 13 to 15 currently using any tobacco products and 22.5% regular smokers.^14^ This puts Timor-Leste as one of the highest current tobacco use rates among adolescents in the world.^7^

The aim of the study was to update estimates of smoking prevalence in school students and identify exposures and factors associated with current smoking in order to inform and direct tobacco control policies in the country.

## Methods

### Study Design and Setting

We carried out a cross-sectional survey of adolescents aged 13 to 15 attending school in the Dili Municipality. Dili is the capital of Timor-Leste and has a population of 230 000 people (approximately 18% of the total population of Timor-Leste) living in six subdistricts.

### Sample Size

The estimated population of students aged 13 to 15 in Dili in 2020 was 17 692. Population proportional to sample size (PPS) was used to calculate the sample size required. To have a 95% confidence level and a 5% margin of error for a prevalence of tobacco use of 25%, a sample size of 285 students was required, and for a 2.5% margin of error, a sample of 1000 students was required. Due to the clustered nature of the sample, these numbers needed to be somewhat larger depending on the degree of clustering of the outcome within schools. We estimated that around 1200 students would give a sufficiently precise estimate of prevalence.

### Sampling Frame and Methods

Schools eligible to be included were *Ensino Básico Central* (EBC, Primary Schools) with students aged 13 (Grade 8) and 14 years (Grade 9), and *Ensino Secúndario Geral* (ESG, Secondary Schools) and *Escola Técnico Vocacional* (ETV Vocational School) schools with students aged 15 years (Grade 10). Students aged 13-15 years were included to replicate earlier studies undertaken in Timor-Leste and in accordance with GYTS guidelines.

A multistage sampling approach was used to select schools and students. A list of eligible schools was obtained from the Office of Education (53 EBCs, 23 ESGs, and 10 ETVs). Twenty percent were selected, which gave 18 schools (12 EBCs and 6 ESGs and ETVs). We ensured all six subdistricts of Dili had schools represented by randomly selecting 12 EBCs with two drawn from each subdistrict. If the selected school agreed for their students to participate, one class from the relevant Grade (approximate class size 50-60 students) was selected randomly at each school. All students who were present on the day of the survey were invited to take part.

### Data Collection

A prepiloted self-completion questionnaire adapted from the 2013 GYTS was used. The questionnaire was translated into Tetum (the local language). Written informed consent was obtained from the school principal of each selected school, and from students in the selected class. The questionnaire was completed in the classroom in the presence of researchers. Data collection was from October 2020 to February 2021.

### Data Variables and Definitions

Data was collected on a range of demographic information, smoking-related knowledge and behaviors, and potential determinants of tobacco use and susceptibility.

Participants were asked about ever and current cigarette smoking, use of other smoked tobacco products (eg, cigars and water pipes) and use of smokeless tobacco products. We also derived combined variables for ever or current use of any form of tobacco product. Ever used was defined as reporting having smoked or used oral tobacco products at least once in their lifetime. Current use was defined as having smoked or used oral tobacco products on one or more days in the past 30 days.

Degree of exposure to smoking in other locations was categorized to exposed on ≥1 day in the last week in none, one, or ≥ 2 locations from answers to three questions about exposure (1) inside any enclosed public place, (2) at any outdoor public place, or (3) inside any public transport. Exposure to smoking messaging in the last month was categorized as none, 1 to 2 media, or ≥3 media from questions about (a) seeing people using tobacco on television, videos, or movies; (b) seeing advertisements or promotions for tobacco at points-of-sale; (c) seeing tobacco brand names at sports events or on television; (d) having something with a tobacco product brand logo; or (e) being offered a free tobacco product. Being taught about the dangers of tobacco use in school in the past 12 months was based on two questions regarding being taught in the classroom or reading in school texts about the health effects of tobacco. Responses to other questions were dichotomized: gender, age (13-14 and 15 years), exposure to smoking in the home in last week (yes/no), exposure to smoking on school premises (≤30 days) (yes/no), belief that smoking is harmful to health (definitely/probably yes vs definitely/probably not), family discussed harmful effects of smoking, and exposure to anti-tobacco messaging (≤3 days) (yes/no). Others were categorized into three levels of exposure: weekly pocket money (none, <US$1, ≥US$1), number of cigarette sellers close to their school (none, 1-2, ≥3), parental smoking (neither, father or mother, father and mother), number of close friends who smoke (none, some, most, or all), and parental employment status (none, one, or both employed).

### Statistical Analysis

All data were entered into an excel spreadsheet, cleaned, and cross-checked. Analysis was undertaken using Stata SE Version 17.0. Data are presented as numbers and proportions with 95% confidence intervals (CIs). We used multivariable logistic regression analysis to investigate possible determinants of current smoking or use of any tobacco products. We report unadjusted odds ratios (OR) and adjusted odds ratios (AOR) from two models—the first adjusted for demographic factors (AOR^1^), and a full model (AOR^2^) which included all potential determinants and confounders. Although strongly associated with current smoking, the number of friends who smoked was not included in the multivariable models because the direction of causality was uncertain. All analyses took account of clustering of data at the level of the individual schools using the *svy* command.

### Ethical Approval

Ethical approval was obtained from the *Insituto Nacional de Saude* (INS), Ministry of Health (Number: 1478/MS-INS/GDE/IX/2020), and the University of Otago Human Ethics Committee (Number: 20/089)

## Results

A total of 1 279 students from 18 schools participated in the study; however, 158 were excluded from the analysis as they were either outside the eligibility age, or their age or sex was not reported. The sociodemographic characteristics of the remaining 1 121 students are shown in Table 1.

**Table 1.** Socio Demographic Characteristics of Participating Students (*n* = 1121).

Characteristic	*n* (%)
Gender	
Male	582 (51.9)
Female	539 (48.1)
Age (years)	
13	192 (17.1)
14	319 (28.5)
15	610 (54.4)
School level	
Primary school	710 (63.3)
Secondary school	337 (30.1)
Vocational school	74 (6.6)
Father’s occupation	
Government employee	319 (28.5)
Private employee	155 (13.8)
Self-employed	401 (35.8)
Unemployed/homemaker	246 (21.9)
Mother’s occupation	
Government employee	126 (11.2)
Private employee	89 (7.9)
Self-employed	330 (29.4)
Unemployed/homemaker	576 (51.4)

### Smoking Prevalence

The prevalence of ever using any form of tobacco product was 40.4% (95% CI [37.5, 43.4]) and was higher in males (55.5%; 95% CI [51.4, 59.6]) than females (23.8%; 95% CI [20.2, 27.6]). The prevalence of current use was 32.2% (95% CI [29.5, 35.1]) and again was higher in males (45.3%; 95% CI [41.2, 49.5]) than females (17.9%; 95% CI [14.7, 21.5]) (Table 2).

**Table 2.** Prevalence of Ever and Current Use of Tobacco Products.

	Ever used/smoked^a^			Current use/smoking (at least once in the past 30 days)^a^		
	Male *n* = 582 % [95% CI]	Female *n* = 539 % [95% CI]	Total *n* = 1121 % [95% CI]	Male *n* = 582 % [95% CI]	Female *n* = 539 % [95% CI]	Total *n* = 1121 % [95% CI]
Cigarette smoking	46.4% [42.3, 50.5]	11.7% [9.1, 14.7]	29.7% [27.0, 32.5]	32.6% [28.8, 36.6]	6.0% [4.0, 8.3]	19.8% [17.5, 22.3]
Other tobacco products	25.0% [21.5, 28.7]	13.1% [10.4, 16.3]	19.3% [17.0, 21.7]	20.7% [17.4, 24.2]	10.3% [7.9, 13.3]	15.7% [13.6, 18.0]
Smokeless tobacco products	16.0% [13.1, 19.3]	12.2% [9.5, 15.3]	14.2% [12.2, 16.4]	13.2% [10.5, 16.3]	11.3% [8.8, 14.3]	12.3% [10.4, 14.4]
Any form of tobacco product	55.5% [51.4, 59.6]	23.8% [20.2, 27.6]	40.4% [37.5, 43.4]	45.3% [41.2, 49.5]	17.9% [14.7, 21.5]	32.2% [29.5, 35.1]

Abbreviation: CI, confidence interval.

a The proportion in each category is of those with complete data (i.e., excludes those with missing or not reported data).

Of the different tobacco products, current cigarette smoking prevalence was highest (19.8%; 95% CI [17.5, 22.3]), followed by other tobacco products (15.7%; 95% CI [13.6, 18.0]), and smokeless tobacco products (12.3%; 95% CI [10.4, 14.4]) (Table 2).

## Prevalence of Exposure to Potential Determinants of Smoking Uptake

Table 3 shows that there was a very high prevalence of exposure to potential determinants of smoking and tobacco product use. For example, almost half (44.7%) of the participants reported that one or more parents smoked, exposure to smoking was very commonly reported in the home (last week, 25.1%), any public place (last week, 40.2%), and schools (last month, 55.6%). Over half (51.6%) reported cigarette sellers close to their school and over two-thirds (66.8%) reported exposure to smoking through TV and other media or to tobacco advertising in the last month. A high proportion (40.4%) reported that smoking was definitely/ probably not harmful to health.

**Table 3.** Factors Associated With Current Cigarette Smoking and Current Use of Any Tobacco Product (at Least Once in the Past 30 Days).

	Current cigarette smoking (*n* = 221/1115)					Current use of any tobacco product (*n* = 351/1089)				
	% exposed	% current smoking	OR [95% CI]	AOR^a^ [95% CI]	AOR^b^ [95% CI]	% exposed	% using any tobacco	OR [95% CI]	AOR^a^ [95% CI]	AOR^b^ [95% CI]
Age (years)										
13-14	45.7	15.3	Ref.	Ref.	Ref.	45.2	26.4	Ref.	Ref.	Ref.
15	54.3	23.6	1.7 [1.2, 2.5]	1.40 [0.9, 2.3]	1.1 [0.8, 1.7]	54.8	37.0	1.6 [1.2, 2.3]	1.4 [1.0, 2.0**]**	1.2 [0.8, 1.7]
Gender										
Female	48.0	6.0	Ref.	Ref.	Ref.	47.8	17.9	Ref.	Ref.	Ref.
Male	52.0	32.6	7.6 [4.4, 13.1]	**7.3 [4.2**, **12.6]**	**7.2 [3.6**, **14.1]**	52.3	45.3	3.8 [2.6, 5.6]	**3.6 [2.4**, **5.4]**	**3.5 [2.3**, **5.2]**
Parents’ employment status										
Both unemployed/homemakers	15.7	17.1	Ref.	Ref.	Ref.	15.7	30.4	Ref.	Ref.	Ref.
One parent employed	42.0	20.3	1.2 [0.7, 2.2]	1.2 [0.7, 2.0]	1.0 [0.6, 1.6]	41.4	32.2	1.1 [0.7, 1.7]	1.1 [0.7, 1.7]	1.0 [0.6, 1.5]
Both parents employed	42.3	20.3	1.2 [0.8, 2.1]	1.2 [0.7, 2.0]	1.0 [0.6, 1.7]	42.9	33.0	1.1 [0.8, 1.7]	1.1 [0.7, 1.6]	1.0 [0.6, 1.5]
Weekly pocket money										
No money	26.9	13.0	Ref.	Ref.	Ref.	27.1	25.1	Ref.	Ref.	Ref.
<US$1	44.4	20.2	1.7 [1.2, 2.5]	**2.1 [1.3**, **3.4]**	**1.8 [1.0**, **3.2]**	44.4	31.0	1.3 [1.0, 1.9]	1.5 [1.0, 2.4]	1.5 [0.9, 2.6]
>US$1	28.7	25.6	2.3 [1.4, 3.9]	**2.6 [1.4**, **4.8]**	**1.9 [1.0**, **3.4]**	28.5	41.0	2.1 [1.4, 3.0]	**2.3 [1.5**, **3.6]**	**2.1 [1.3**, **3.5]**
Closest friends smoke tobacco^c^										
None	82.1	15.7	Ref.	Ref.	—	82.0	28.0	Ref.	Ref.	—
Some	9.3	29.4	2.2 [1.3, 3.8]	1.8 [0.9, 3.3]	—	9.3	42.0	1.9 [1.2, 2.8]	**1.5 [1.0, 2.4]**	—
Most or all	8.6	48.9	5.2 [3.2, 8.3]	**4.3 [2.3, 8.2]**	—	8.7	60.2	3.9 [2.6, 5.9]	**3.3 [1.9, 5.6]**	—
Parents smoke										
Neither	55.4	17.2	Ref.	Ref.	Ref.	55.3	28.3	Ref.	Ref.	Ref.
Father or mother	39.2	19.9	1.2 [0.8, 1.8]	1.2 [0.8, 1.9]	1.1 [0.6, 2.0]	39.1	32.0	1.2 [1.0, 1.5]	1.2 [1.0, 1.6]	1.2 [0.8, 1.6]
Father and mother	5.3	33.9	2.5 [1.3, 4.7]	2.1 [0.9, 4.5]	1.5 [0.6, 3.6]	5.6	57.9	3.5 [2.2, 5.6]	**3.1 [1.7**, **5.9]**	**3.4 [1.5**, **7.3]**
Exposure to smoking in the home in the last week										
No	75.5	12.9	Ref.	Ref.	Ref.	74.9	25.3	Ref.	Ref.	Ref.
Yes	24.5	41.2	4.7 [2.9, 7.7]	**5.0 [3.0**, **8.3]**	**2.4 [1.4**, **4.2]**	25.1	52.8	3.3 [2.3, 4.7]	**3.2 [2.3**, **4.5]**	**2.1 [1.3**, **3.3]**
Exposure to smoking in other locations (public transport, enclosed public or outdoor public place) in the last week										
None	59.7	9.9	Ref.	Ref.	Ref.	59.7	21.5	Ref.	Ref.	Ref.
1 place SHS exposure	17.6	23.8	2.8 [1.9, 4.3]	**2.7 [1.8**, **4.2]**	**1.6 [1.0**, **2.7]**	17.4	42.3	2.7 [1.8, 3.9]	**2.6 [1.8**, **3.7]**	**2.1 [1.3**, **3.2]**
2+ place SHS exposures	22.7	42.7	6.8 [4.0, 11.6]	**6.6 [3.7**, **11.6]**	**3.0 [1.7**, **5.4]**	22.8	53.1	4.1 [2.8, 6.1]	**3.8 [2.6**, **5.5]**	**2.0 [1.2**, **3.2]**
Exposure to smoking in the school environment in the last month										
No	44.3	17.0	Ref.	Ref.	Ref.	44.4	26.6	Ref.	Ref.	Ref.
Yes	55.7	21.7	1.4 [1.0, 1.9]	1.4 [1.0, 2.1]	0.9 [0.5, 1.5]	55.6	36.5	1.6 [1.2, 2.1]	**1.7 [1.2**, **2.3]**	1.2 [0.8, 1.8]
There are cigarette sellers close to the school										
None	48.4	15.5	Ref.	Ref.	Ref.	48.4	30.5	Ref.	Ref.	Ref.
One or two	38.7	21.7	1.5 [1.0, 2.3]	**1.6 [1.0**, **2.3]**	0.9 [0.5, 1.6]	38.9	31.9	1.1 [0.7, 1.5]	1.1 [0.7, 1.6]	0.6 [0.4, 1.0]
Three or more	13.0	29.6	2.3 [1.4, 3.6]	**2.6 [1.6**, **4.3]**	1.2 [0.8, 1.7]	12.7	39.7	1.5 [1.0, 2.3]	**1.6 [1.0**, **2.5]**	0.7 [0.4, 1.2]
Exposure to smoking messaging through television, advertisements, and other media in the last month										
No	33.5	11.5	Ref.	Ref.	Ref.	33.2	19.9	Ref.	Ref.	Ref.
1-2 media	42.0	20.0	1.9 [1.2, 3.0]	**2.0 [1.3**, **3.2]**	1.2 [0.7, 2.4]	42.3	34.2	2.1 [1.4, 3.2]	**2.2 [1.4**, **3.4]**	1.3 [0.7, 2.3]
3+ media	24.5	32.3	3.7 [2.1, 6.4]	**3.4 [2.0**, **5.8]**	1.7 [0.9, 3.3]	24.5	46.2	3.5 [1.0, 6.0]	**3.1 [1.9**, **5.3]**	1.7 [0.9, 3.3]
Believe that smoking is harmful to my health										
Definitely/probably yes	59.6	21.0	Ref.	Ref.	Ref.	59.6	30.9	Ref.	Ref.	Ref.
Definitely/probably not	40.4	18.0	0.8 [0.6, 1.2]	0.7 [0.5, 1.1]	1.2 [0.7, 1.9]	40.4	34.0	1.1 [0.9, 1.5]	1.1 [0.9, 1.4]	**2.0 [1.4**, **2.8]**
Have been taught about the dangers of tobacco use in school in the past 12 months										
No	50.1	15.7	Ref.	Ref.	Ref.	50.1	26.6	Ref.	Ref.	Ref.
Yes	49.9	24.2	1.7 [1.1, 2.7]	1.6 [1.0, 2.7]	1.0 [0.6, 1.7]	49.9	38.3	1.7 [1.2, 2.5]	**1.7 [1.1**, **2.6**]	1.1 [0.7, 1.8]
Family have discussed the harmful effects of smoking										
No	57.9	14.3	Ref.	Ref.	Ref.	58.0	24.4	Ref.	Ref.	Ref.
Yes	42.1	27.4	2.3 [1.8, 3.0]	**2.0 [1.5**, **2.8]**	1.4 [0.9, 2.2]	42.0	43.2	2.4 [1.8, 3.1]	**2.2 [1.5**, **3.0]**	**1.6 [1.1**, **2.3]**
Have been exposed to anti-tobacco messaging in the last month										
No	15.2	14.4	Ref.	Ref.	Ref.	15.5	19.9	Ref.	Ref.	Ref.
Yes	84.8	20.7	1.6 [0.9, 2.9]	1.7 [0.8, 3.4]	1.0 [0.4, 2.6]	84.5	34.4	2.1 [1.2, 3.7]	**2.3 [1.2**, **4.3]**	1.7 [0.8, 3.8]

Abbreviations: AOR, adjusted odds ratio; CI, confidence interval; OR, odds ratio; SHS, second hand smoke.

a AOR for age, gender, and parents employment.

b Fully AOR with all variables included in the model, including the school to account for clustering.

c Not included in the fully adjusted model due to uncertainty about the direction of causality.

Bold values indicate the results that were statistically significant.

### Factors Associated With Current Cigarette Smoking and Use of Any Tobacco Product

Table 3 shows the findings from the univariate, minimally adjusted, and fully adjusted models. After including all variables (except friends smoking) in the model, only being male (AOR^2^ 7.2; 95% CI [3.6, 14.1]), exposure to smoking in the home (AOR^2^ 2.4; 95% CI [1.4, 4.2]), and exposure to smoking in other public places (AOR^2^ 3.0; 95% CI [1.7, 5.4]) remained significantly associated with current cigarette smoking.

For current use of a tobacco product in the fully adjusted model, being male (AOR^2^ 3.5; 95% CI [2.3, 5.2]), having ≥US$1 weekly pocket money (AOR^2^ 2.1; 95% CI [1.3, 3.5]), both parents smoking (AOR^2^ 3.4; 95% CI [1.5, 7.3]), exposure to smoking in the home (AOR^2^ 2.1; 95% CI [1.3, 3.3]), exposure to smoking in other locations (1 place: AOR^2^ 2.1; 95% CI [1.3, 3.2]; 2+ places: AOR^2^ 2.0; 95% CI [1.2, 3.2]), and family discussing the harmful effects of smoking (AOR^2^ 1.6; 95% CI [1.1, 2.3]) remained statistically significant (Table 3).

## Cigarette Purchasing By Current Smokers

Of the students who had smoked a cigarette in the past 30 days and provided an answer to the question about place of purchase (143/221 students), half (51%) bought cigarettes from a kiosk, 10% from a street vendor, 11% from a shop or store, and 27% obtained the cigarette/s from someone else (eg, a friend or family member). When asked about how they bought them, 39% (of 128 who completed this question) bought cigarettes as single/s, 33% bought them in a pack, 6% in a carton, 13% bought tobacco and rolled their own, and for 9% the purchase means was not specified. Sixty-two percent (77 of 124) has been refused the sale of a cigarette because of their age.

## Discussion

Our study adds to earlier evidence showing that the prevalence of smoking and any tobacco product use in young adolescents, particularly boys, remains unacceptably high in Timor-Leste and hence is a key public health issue. Risk factors for smoking and any tobacco product use included parental smoking, exposure to smoking in the home and other public locations, exposure to smoking in the media or messaging about smoking, and having access to pocket money. Exposure to these, and other potential determinants of smoking and tobacco use, was very common suggesting that despite legislation being in place, much more could be done at a national and local level to enforce existing legislation and introduce new policy measures and other health promotion interventions to help protect adolescents from becoming tobacco users.

The proportion of students who reported current smoking of cigarettes (19.8%) or any tobacco product (32.2%) in our study was similar to that reported in the 2019 GYTS in Timor-Leste (20.4% and 30.9%, respectively) which is also a school-based survey in the same age group but with students recruited from throughout the country.^14^ What is encouraging to note is the lower prevalence in both our study and the 2019 GYTS compared with the 2013 GYTS which reported 28.9% current cigarette smoking and 42.4% smoking any tobacco product.^9^

However, compared with other countries in the region and indeed in the world, adolescent smoking in Timor-Leste remains among the highest.^7,15-18^ The average prevalence of current tobacco use in the Southeast Asian region is 9.3% (12.3% in boys and 6.1% in girls) and current cigarette smoking is 5.6% (9.2% in boys and 1.8% in girls).^8^ Timor-Leste appears to be even higher than its neighboring country, Indonesia (2019 current smoking of any tobacco product = 19.2% (35.6% boys; 3.5% girls)), which has not yet ratified the FCTC.^19^ It is concerning to note the increase in use of smokeless tobacco from 8.4% in the 2013 GYTS^9^ to 13.9% in the 2019 GYTS^14^ and 12.3% in our study. These rates also appear to be considerably higher than many other countries (global prevalence 3.2% boys; 1.9% girls).^8^

Some of the reasons for the continued high prevalence of smoking in Timor-Leste may be found in the high levels of exposure to potential determinants identified in our study. Having access to cigarettes to three or more sellers close to the school, which has previously been found to be associated with adolescent smoking,^20,21^ was associated with smoking and tobacco use in the minimally adjusted model but not in the fully adjusted model. This may be due to the widespread and easy availability of cigarettes throughout Dili from street vendors and kiosks, as well as the ability to purchase single cigarettes—despite this being illegal—therefore the vendor being close to the school is of less relevance.^22^ The whole picture showing ready availability of tobacco products is an important issue needing to be addressed to help reduce adolescent smoking in Timor-Leste. The recent increase in excise tax on tobacco products from US$50 to US$100 per kilogram shows a commitment of the government to deal with tobacco consumption.^23,24^

While Timor-Leste has implemented some legislation and public health interventions to prevent smoking, the findings suggest there continues to be easy access to cigarettes, minimal regulation of smoking in public places, and widespread tobacco advertising through different modes. Students in our study had an increased risk of being current smokers if they had been exposed to smoking in the home and in public places, exposed to tobacco messaging, or had pocket money available to spend, all of which are consistent with findings from other studies.^16,17,25-28^ Community-based health promotion interventions to discourage smoking around children especially in the home and encourage parents to monitor children’s pocket money expenditure may be needed, together with stronger legislation and enforcement of regulations such as prohibitions on selling cigarettes to minors or as single cigarettes, restrictions on the number of tobacco retailers (especially street vendors and kiosks), banning smoking in public places and in schools and further restrictions on tobacco related advertising. Parental smoking was associated with smoking and with use of any tobacco product, and was identified as a determinant of smoking initiation in a systematic review.^29^ The association of parental smoking with adolescent tobacco use and the high rates of adult smoking in Timor-Leste suggest that increased support for adults to quit (particularly parents) and stronger tobacco control policy and legislation to reduce adult smoking prevalence may also be needed to help reduce adolescent smoking.

The higher rates amongst males in our study are also consistent with other studies.^16,30^ While this could indicate a need for specific targeting of prevention programs toward males, it still needs to be noted that the prevalence of current use of any form of tobacco product among females is high at 17.9% and ever use even higher at 23.8%. This again points to the need for national regulations and enforcement which will impact on reducing smoking in the overall population—including adults.

It is not clear why, in the fully adjusted model, students whose family had discussed the harmful effects of smoking were more likely to be current smokers. However, this may be due to reverse causation with family discussions (or recall of discussions) of smoking harms being more likely when children were smoking. Moreover, exposure to anti-tobacco messaging and being taught of the dangers of smoking in school were also associated with smoking in the minimally adjusted, but not the fully adjusted model. Again, this may be because students who smoke pay more attention to, or recall, these messages more clearly, rather than anti-tobacco messaging increasing the risk of smoking. In a cross-sectional study, we can only establish associations and not the direction of association.

The strengths of our study include the random selection of schools and the relatively large sample size and wide range of data collected on smoking and related factors. Weaknesses included the self-reported nature of the data (e.g., there was no biochemical validation of smoking status) and possible occurrence of nonreporting which may have underestimated smoking or tobacco use prevalence. Moreover, establishing the causal directions of potential determinants of smoking and tobacco use are not possible due to the cross-sectional design of the study.

## Conclusion

As we have shown, there is a complex array of different factors at different levels impacting on adolescent smoking which need to be dealt with to see any shift in smoking prevalence and behaviors. Specifically, a renewed commitment to the Dili Declaration^12^ is required and policymakers across the various government ministries such as the Ministry of Health, Ministry of Education, Youth and Sport, and Ministry of Finance, need to be made aware of these results and stronger enforcement of legislation actioned and new policy measures considered. Reducing exposure to smoking in the home is also likely to be key^27^ and possibilities to implement awareness campaigns need to be explored.
